# *RAB32* Ser71Arg in autosomal dominant Parkinson's disease: linkage, association, and functional analyses

**DOI:** 10.1016/S1474-4422(24)00121-2

**Published:** 2024-06

**Authors:** Emil K Gustavsson, Jordan Follett, Joanne Trinh, Sandeep K Barodia, Raquel Real, Zhiyong Liu, Melissa Grant-Peters, Jesse D Fox, Silke Appel-Cresswell, A Jon Stoessl, Alex Rajput, Ali H Rajput, Roland Auer, Russel Tilney, Marc Sturm, Tobias B Haack, Suzanne Lesage, Christelle Tesson, Alexis Brice, Carles Vilariño-Güell, Mina Ryten, Matthew S Goldberg, Andrew B West, Michele T Hu, Huw R Morris, Manu Sharma, Ziv Gan-Or, Bedia Samanci, Pawel Lis, Maria Teresa Periñan, Rim Amouri, Samia Ben Sassi, Faycel Hentati, Global Parkinson's Genetics Program (GP2), Francesca Tonelli, Dario R Alessi, Matthew J Farrer

**Affiliations:** a Department of Genetics and Genomic Medicine, Great Ormond Street Institute of Child Health, University College London, London, UK; b Department of Clinical and Movement Neurosciences, UCL Queen Square Institute of Neurology, University College London, London, UK; c UCL Movement Disorders Centre, University College London, London, UK; d NIHR Great Ormond Street Hospital Biomedical Research Centre, University College London, London, UK; e McKnight Brain Institute, Department of Neurology, University of Florida, Gainesville, FL, USA; f Institute of Neurogenetics, University of Lübeck, Lübeck, Germany; g Department of Neurology, Center for Neurodegeneration and Experimental Therapeutics, University of Alabama at Birmingham, Birmingham, AL, USA; h Department of Medical Genetics, University of British Columbia, Vancouver, BC, Canada; i Pacific Parkinson's Research Centre, Djavad Mowafaghian Centre for Brain Health, Division of Neurology, University of British Columbia, Vancouver, BC, Canada; j Movement Disorders Program, Division of Neurology, University of Saskatchewan and Saskatchewan Health Authority, Saskatoon, SK, Canada; k Department of Pathology, University of Saskatchewan and Saskatchewan Health Authority, Saskatoon, SK, Canada; l Institute for Medical Genetics and Applied Genomics, University of Tübingen, Tübingen, Germany; m Centre for Genetic Epidemiology, Institute for Clinical Epidemiology and Applied Biometry, University of Tübingen, Tübingen, Germany; n Sorbonne Université, Institut du Cerveau–Paris Brain Institute–ICM, Inserm, CNRS, Paris, France; o Assistance Publique Hôpitaux de Paris, Hôpital Pitié-Salpêtrière, Département de Neurologie, Centre d'Investigation Clinique Neurosciences, DMU Neuroscience, Paris, France; p Duke Center for Neurodegeneration and Neurotherapeutics, Department of Pharmacology and Cancer Biology, Duke University, Durham, NC, USA; q Division of Neurology, Nuffield Department of Clinical Neurosciences, University of Oxford, Oxford, UK; r The Neuro, Montreal Neurological Institute-Hospital, Montreal, QC, Canada; s Department of Neurology and Neurosurgery, and Department of Human Genetics, McGill University, Montreal, QC, Canada; t Behavioural Neurology and Movement Disorders Unit, Department of Neurology, Istanbul Faculty of Medicine, Istanbul University, Istanbul, Turkey; u MRC Protein Phosphorylation and Ubiquitylation Unit, School of Life Sciences, University of Dundee, Dundee, UK; v Aligning Science Across Parkinson's Collaborative Research Network, Chevy Chase, MD, USA; w Queen Mary College, University of London, London, UK; x Service de Neurologie, Institut National de Neurologie, La Rabta, Tunis, Tunisia

## Abstract

**Background:**

Parkinson's disease is a progressive neurodegenerative disorder with multifactorial causes, among which genetic risk factors play a part. The RAB GTPases are regulators and substrates of LRRK2, and variants in the *LRRK2* gene are important risk factors for Parkinson's disease. We aimed to explore genetic variability in RAB GTPases within cases of familial Parkinson's disease.

**Methods:**

We did whole-exome sequencing in probands from families in Canada and Tunisia with Parkinson's disease without a genetic cause, who were recruited from the Centre for Applied Neurogenetics (Vancouver, BC, Canada), an international consortium that includes people with Parkinson's disease from 36 sites in 24 countries. 61 RAB GTPases were genetically screened, and candidate variants were genotyped in relatives of the probands to assess disease segregation by linkage analysis. Genotyping was also done to assess variant frequencies in individuals with idiopathic Parkinson's disease and controls, matched for age and sex, who were also from the Centre for Applied Neurogenetics but unrelated to the probands or each other. All participants were aged 18 years or older. The sequencing and genotyping findings were validated by case–control association analyses using bioinformatic data obtained from publicly available clinicogenomic databases (AMP-PD, GP2, and 100 000 Genomes Project) and a private German clinical diagnostic database (University of Tübingen). Clinical and pathological findings were summarised and haplotypes were determined. *In-vitro* studies were done to investigate protein interactions and enzyme activities.

**Findings:**

Between June 1, 2010, and May 31, 2017, 130 probands from Canada and Tunisia (47 [36%] female and 83 [64%] male; mean age 72·7 years [SD 11·7; range 38–96]; 109 White European ancestry, 18 north African, two east Asian, and one Hispanic] underwent whole-exome sequencing. 15 variants in RAB GTPase genes were identified, of which the *RAB32* variant c.213C>G (Ser71Arg) cosegregated with autosomal dominant Parkinson's disease in three families (nine affected individuals; non-parametric linkage Z score=1·95; p=0·03). 2604 unrelated individuals with Parkinson's disease and 344 matched controls were additionally genotyped, and five more people originating from five countries (Canada, Italy, Poland, Turkey, and Tunisia) were identified with the *RAB32* variant. From the database searches, in which 6043 individuals with Parkinson's disease and 62 549 controls were included, another eight individuals were identified with the *RAB32* variant from four countries (Canada, Germany, UK, and USA). Overall, the association of *RAB32* c.213C>G (Ser71Arg) with Parkinson's disease was significant (odds ratio [OR] 13·17, 95% CI 2·15–87·23; p=0·0055; *I*^2^=99·96%). In the people who had the variant, Parkinson's disease presented at age 54·6 years (SD 12·75, range 31–81, n=16), and two-thirds had a family history of parkinsonism. *RAB32* Ser71Arg heterozygotes shared a common haplotype, although penetrance was incomplete. Findings in one individual at autopsy showed sparse neurofibrillary tangle pathology in the midbrain and thalamus, without Lewy body pathology. In functional studies, RAB32 Arg71 activated LRRK2 kinase to a level greater than RAB32 Ser71.

**Interpretation:**

*RAB32* Ser71Arg is a novel genetic risk factor for Parkinson's disease, with reduced penetrance. The variant was found in individuals with Parkinson's disease from multiple ethnic groups, with the same haplotype. In-vitro assays show that RAB32 Arg71 activates LRRK2 kinase, which indicates that genetically distinct causes of familial parkinsonism share the same mechanism. The discovery of *RAB32* Ser71Arg also suggests several genetically inherited causes of Parkinson's disease originated to control intracellular immunity. This shared aetiology should be considered in future translational research, while the global epidemiology of *RAB32* Ser71Arg needs to be assessed to inform genetic counselling.

**Funding:**

National Institutes of Health, the Canada Excellence Research Chairs program, Aligning Science Across Parkinson's, the Michael J Fox Foundation for Parkinson's Research, and the UK Medical Research Council.

**Research in context**

**Evidence before this study**

We searched PubMed with the search terms “Parkinson's disease”, “Parkinsonism” and “RAB32” for articles published in English on or before Jan 4, 2024, in any field. We found a total of seven articles. No studies were found describing genetic variability in *RAB32* in Parkinson's disease or parkinsonism. One epigenome-wide study of CD14+ monocytes suggests hypomethylation and increased expression of *RAB32* is associated with idiopathic Parkinson's disease. Altogether, the articles serve to highlight an interaction between LRRK2 and RAB32, which potentially coordinates a cell-intrinsic itaconate-dependent defence pathway against intracellular pathogens. A similar search for “RAB32” and “PINK1” revealed no results. However, PINK1 is well described in mitochondrial quality control and mitophagy, and this process might restrict inflammatory cytokine secretion and directly regulate antigen presentation and immune cell homoeostasis.

**Added value of this study**

We have shared our discovery of *RAB32* Ser71Arg in prior conference proceedings and a review hoping more affected probands and families would be identified. However, through genetic linkage and association meta-analysis, this study is the first to provide strong evidence to suggest that *RAB32* Ser71Arg causes familial parkinsonism. We demonstrate that RAB32 Ser71Arg activates LRRK2 kinase, we show that it colocalises with PINK1, and we highlight its association with retromer (VPS35 via VARP). Hence, RAB32 is a nexus that unites at least four major gene discoveries for familial and seemingly sporadic Parkinson's disease. Moreover, our data suggest that a convergence of evolution underlies this molecular mechanism for disease pathogenesis.

**Implications of all the available evidence**

Our study provides the first evidence for *RAB32* Ser71Arg as a cause of late-onset Parkinsonism. The Ser71Arg mutation seems to originate from one founder despite its worldwide diaspora, and appears to segregate with disease, albeit with reduced penetrance. Functional analysis shows that RAB32 Arg71 activates LRRK2 kinase to a level greater than RAB32 Ser71 (wild-type) without disrupting the interaction between these two proteins. *RAB32* Ser71Arg also reduced colocalisation with the mitochondrial kinase PINK1. Mutations in LRRK2 that lead to constitutive activation of its kinase domain are genetically linked to Parkinson's disease and are associated with mitochondrial health and impaired mitophagy. Our study provides a unique, genetically stratified cause of Parkinson's disease that is distinct from *LRRK2* and is likely to both benefit from and provide unmet insight into LRRK2 function and inhibitor testing within the clinic. As LRRK2 kinase inhibitors are being trialled as a disease-modifying therapy in Parkinson's disease, further work is needed to establish their potential in *RAB32* Ser71Arg Parkinson's disease.

## Introduction

Parkinson's disease is a progressive neurodegenerative disease that can have many causes. This movement disorder is invariably associated with a loss of dopaminergic neurons in the substantia nigra pars compacta. To date, 19–37% of the genetic heritability of Parkinson's disease has been accounted for, and efforts are ongoing to identify novel Parkinson's disease loci and pathogenic variants.^1^ Thus far, Mendelian genetic discoveries have highlighted autophagic (lysosomal), mitochondrial, and endosomal proteins that are implicated in the disease. For example, pathogenic variants have been identified in *SNCA* in families with dominantly inherited parkinsonism.^2^, ^3^ Monomeric α-synuclein (which is encoded by *SNCA*) functions as a vesicular chaperone,^4^ but it can aggregate into Lewy body pathology,^5^ which is pathognomonic for Parkinson's disease.^4^ Additionally, *PRKN* and *PINK1* mutations were discovered in young people with recessively inherited Parkinson's disease, and variants in these genes highlight deficits in mitochondrial function, mitophagy, and intracellular immunity.^6^, ^7^, ^8^ A pathogenic variant in *VPS35* (c.1858G>A; Asp620Asn; NCBI accession number NM_018206.4) also leads to dominantly inherited parkinsonism. VPS35 is part of the retromer complex that enables cargo recycling of endosomal membrane-associated proteins, including the dopamine transporter.^9^ Finally, pathogenic variants in *LRRK2* cause dominantly inherited parkinsonism through augmented kinase activity. LRRK2 phosphorylates several RAB GTPase molecules at a conserved residue in the switch II domain to control effector binding activities, localisation, and function,^10^ and a subset of RAB GTPases are genetically implicated in idiopathic Parkinson's disease and atypical parkinsonism.^1^, ^11^, ^12^

The RAB family comprises 61 small GTPases that all function as molecular switches to regulate intracellular vesicular trafficking, alternating between two conformational states—the GTP-bound on form, and the GDP-bound off form. We postulated that additional RAB GTPases might be genetically linked to familial parkinsonism or associated with idiopathic Parkinson's disease, or both. We aimed to assess genetic variability in RAB GTPase genes using whole-exome sequencing (WES) and segregation analysis in families with multi-incident Parkinson's disease, with validation through case–control association analyses and functional studies.

## Methods

### Participants

We recruited probands and asymptomatic family members from the Centre for Applied Neurogenetics, which was established through a Canada Excellence Research Chair (MJF). This 7-year award, based at the University of British Columbia (Vancouver, BC, Canada), enabled an international network for research into Parkinson's disease. Probands and their families from 36 sites in 24 countries were included in the network, and recruitment was done between July 1, 2010, and June 30, 2017. All sites within the network obtained local ethics committee approval before participant recruitment. Within the network, all probands and asymptomatic family members were examined by neurologists with expertise in movement disorders, generally as part of their clinical care (appendix 1 p 2). Affected probands and their families provided written informed consent to participate in this clinicogenetic research, as well as blood or DNA samples. Probands were diagnosed with Parkinson's disease according to UK Brain Bank Criteria,^13^ which had been modified to allow for a positive family history of Parkinson's disease. For the purposes of this study, we only included probands from Canada and Tunisia who were from pedigrees with multi-incident parkinsonism (ie, two or more family members diagnosed as having Parkinson's disease or primary parkinsonism) in whom no known Mendelian causes of Parkinson's disease had been identified. Neuropathological analyses were performed in accordance with biomedical research ethics procedures at the University of Saskatchewan (Saskatoon, SK, Canada; appendix 1 p 3).

### Procedures

To explore the genetic variability of RAB GTPases in Parkinson's disease, probands were screened for known pathogenic variants in Parkinson's disease-related genes by WES (appendix 1 p 2); asymptomatic family members were not included in this screening process. All variants observed were assigned a Genomic Evolutionary Rate Profiling scores (GERP; range –12·3 to 6·17, with 6·17 being the most conserved) and Combined Annotation Dependent Depletion values (CADD; a compositive prediction of how deleterious a nucleotide variant is in the human genome, range 1–99. A CADD≥20 is the top 1% of the most deleterious variants). Public references were also used to provide minor allele frequencies (MAF) including the NCBI Allele Frequency Aggregator dataset (ALFA) and the Genome Aggregation Database (gnomAD version 4.0.0.0).

Putatively pathogenic variants in RAB GTPases were ranked by their rarity, high GERP and CADD scores, then analysed for segregation with Parkinson's disease within the multi-incident families in which they were identified. Top candidates—ie, those variants in RAB genes that were identified in more than one family—were genotyped in additional series of people with Parkinson's disease at the Centre for Applied Neurogenetics (who were unrelated to the probands and to each other), and controls who were matched by country of origin, age, and sex. Subsequent, large-scale screening of the single most promising *RAB* gene and variant was bioinformatically performed using whole-genome sequencing (WGS) data from three publicly available clinicogenomic databases—Accelerating Medicines Partnership in Parkinson's Disease (AMP-PD), Global Parkinson's Genetics Program (GP2), and the 100 000 Genomes Project—and a privately owned German clinical diagnostic database (University of Tübingen, Tübingen, Germany; appendix 2 p 3).^14^

**Figure 1:**
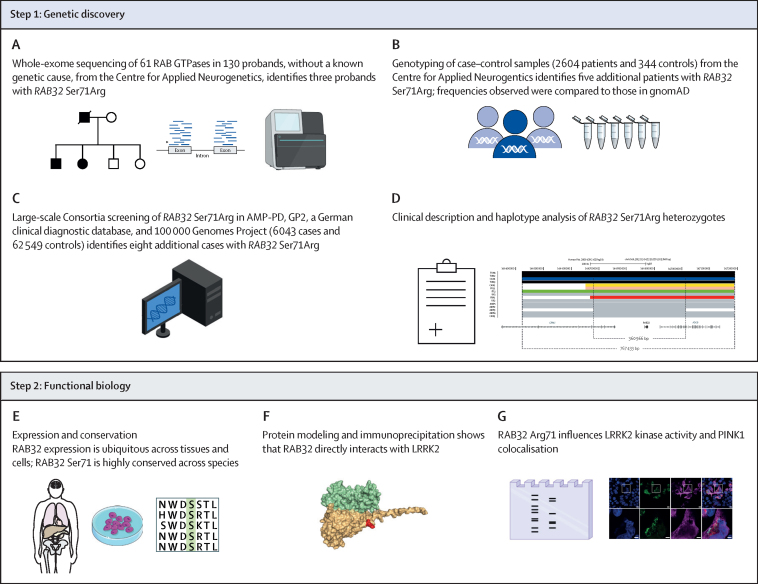
Schematic outline of the methodological framework used in the study AMP-PD=Accelerating Medicines Partnership in Parkinson's Disease. GP2=Global Parkinson's Genetics Program.

To assess whether candidate RAB variants shared a common ancestral haplotype, single nucleotide polymorphisms (SNPs) were genotyped in the pedigrees (figure 1D; appendix 1 p 3). Gametic phase was assessed within pedigrees. Genotypes for the same SNPs were retrieved from whole genome data within public databases as a confirmatory step, using bcftools (release 1.18).

Functional biology included assessment of mRNA and protein expression of the candidate RAB using tissue samples obtained from publicly available datasets. mRNA expression was assessed using Genotype-Tissue Expression (GTEx version 8, that includes 54 different human tissues; Broad Institute of MIT and Harvard) and protein expression was assessed with the Human Protein Atlas (that includes 44 normal human tissue types; Knut & Alice Wallenberg Foundation; appendix 1 p 3). Furthermore, we did immunohistochemical staining in C57BL/6 mice (JAX stock #000664) to assess candidate protein expression in the substantia nigra pars compacta (appendix 1 pp 4–5).

To predict the potential effect of the candidate RAB variant on LRRK2 protein, structural modelling was done with AlphaFold (Google DeepMind and the European Molecular Biology Laboratory–European Bioinformatic Institute; appendix 1 p 3).^15^ Resulting structures were analysed using PyMOL 2.5.5. (Informer Technologies) BIOVIA Discovery Studio Visualizer 2021 (Dassault Systemes) was used to determine possible intermolecular interactions (ie, the non-covalent interactions between atoms) and to predict potential rotamers (ie, that represent the local energy minima and ranges of torsional angles for amino acid side chains in the protein model).

Quantitative western blotting was done in HEK293 cells to assess the potential relationship between LRRK2 activity and the candidate protein variant (appendix 1 p 4). RAB29 was used as a positive control as it is known to induce activation of LRRK2.^16^ We also treated cells with the small molecule kinase inhibitor (MLi-2) to inhibit LRRK2 kinase activity. Densitometric signals from western blots were analysed in ImageJ software.^17^ Several RAB GTPases are known to activate and be substrates of LRRK2. Therefore, we used two assays to determine whether the candidate variant would change the interaction between the mutated RAB protein and LRRK2 and cause activation of LRRK2 kinase. An in vitro cell system^18^ tested the interaction between LRRK2 and the mutated RAB32 protein whereas phosphorylation sites known to correlate with LRRK2 activity (LRRK2-Ser1292 [autophosphorylation site],^19^ LRRK2-Ser935 [biomarker site], and a LRRK2-direct substrate [RAB10-Thr73])^10^ provided an in-vitro assessment of LRRK2 kinase activity.

### Statistical analysis

To assess segregation of the candidate variant with Parkinson's disease, non-parametric linkage analysis (Whittemore and Halpern) was done in multi-incident families, using the Kong and Cox 1997 exponential model, as implemented by Merlin 1.1.2 (Abecasis Lab, School of Public Health, University of Michigan, Ann Arbor, MI, USA).^20^ Association analyses were done to examine the association of candidate variant with Parkinson's disease risk in each case–control series, using Fisher's exact test and random-effects meta-analysis modelling, with the metafor package for R.^21^ A p value ≤0·05 was considered statistically significant in single variant analyses, and in ANOVA, prior to performing post-hoc Tukey's tests adjusted for multiple comparisons.

### Role of the funding source

The funders of the study had no role in study design, data collection, data analysis, data interpretation, or writing of the report.

## Results

130 probands from pedigrees with multi-incident parkinsonism were included, of whom 47 were female and 83 were male, mean age at onset was 54·2 years (SD 14·1; range 13–83), and 109 were White, 18 were of north African ancestry, two were east Asian, and one was Hispanic (table 1). No known Mendelian causes of Parkinson's disease were found. Probands underwent WES and RAB GTPase variant analysis in 61 RAB GTPase genes (figure 1A; appendix 2 p 1). 15 heterozygous rare (gnomAD v4.0 MAF<0·001) and putatively damaging non-synonymous variants in RAB GTPase genes were reported (table 2).

**Table 1:** Clinical characteristics of 130 probands with Parkinson's disease without a known genetic cause, from the Centre for Applied Neurogenetics

	**Probands (n=130)**
**Sex**	
Female	47 (36%)
Male	83 (64%)
**Age at onset, years**	
Mean	54·2 (14·1)
Range	13–83
**Age at study, years**	
Mean	72·7 (11·7)
Range	38–96
**Race**	
White	109
East Asian	2
Hispanic	1
North African	18

**Table 2:** 15 rare non-synonymous variants in the RAB GTPase genes in 130 probands with Parkinson's disease or primary parkinsonism

	**Chr:position (*Human reference genome*/GRCh37 assembly, *hg19*)**	**Reference/Alternate allele**	**NCBI Reference Sequence**	**Coding nucleotide change**	**Amino acid change**	**Reference SNP identifier**	**Allele frequency (gnomAD v4.0)**	**CADD phred-like score**	**Number of carriers (from 130 probands)**	**Zygosity**
*RAB3B*	1:52385700	T/C	NM_002867	559A>G	Met187Val	rs34017695	6·90 × 10^−4^	27·8	1	Heterozygote
*RAB17*	2:238483760	G/T	NM_022449	541C>A	Leu181Met	rs112742374	1·75 × 10^−4^	35·0	1	Heterozygote
*RAB6B*	3:133553479	G/A	NM_016577	502C>T	Arg168Ter	rs147187493	2·48 × 10^−6^	44·0	1	Heterozygote
*RAB28*	4:13373484	A/C	NM_001159601	581T>G	Phe194Cys	rs1560264563	2·16 × 10^−6^	25·0	1	Heterozygote
*RAB24*	5:176729484	T/C	NM_001031677	347A>G	Tyr116Cys	rs1475147714	6·57 × 10^−6^	23·4	1	Heterozygote
*RAB32*	6:146865220	C/G	NM_006834	213C>G	Ser71Arg	rs200251693	9·30 × 10^−6^	19·2	3	Heterozygote
*RAB38*	11:87908390	G/A	NM_022337	163C>T	Pro55Ser	rs769358206	2·74 × 10^−6^	35·0	1	Heterozygote
*RAB27A*	15:55497812	G/A	NM_004580	559C>T	Arg187Trp	rs144946000	1·41 × 10^−4^	24·5	1	Heterozygote
*RAB27A*	15:55516087	C/G	NM_004580	467G>C	Gly156Ala	rs200031368	5·83 × 10^−5^	41·0	1	Heterozygote
*RAB8B*	15:63548776	A/T	NM_016530	397A>T	Lys133Ter	NA	NA	19·0	1	Heterozygote
*RAB26*	16:2201727	G/A	NM_014353	380G>A	Arg127H	rs149935646	2·27 × 10^−4^	34·0	1	Heterozygote
*RAB37*	17:72733173	G/A	NM_001163989	25G>A	Gly9Arg	rs762798603	1·62 × 10^−5^	16·5	1	Heterozygote
*RAB40B*	17:80616378	C/T	NM_006822	554G>A	Arg185Gln	rs199886901	4·22 × 10^−4^	13·7	1	Heterozygote
*RAB12*	18:8636320	A/G	NM_001025300	586A>G	Ile196Val	rs143888944	5·26 × 10^−4^	19·12	1	Heterozygote
*RAB36*	22:23503707	G/A	NM_004914	958G>A	Glu320Lys	rs9624038	3·94 × 10^−4^	33·0	1	Heterozygote

CADD=Combined Annotation Dependent Depletion. NA=not available

**Figure 2:**
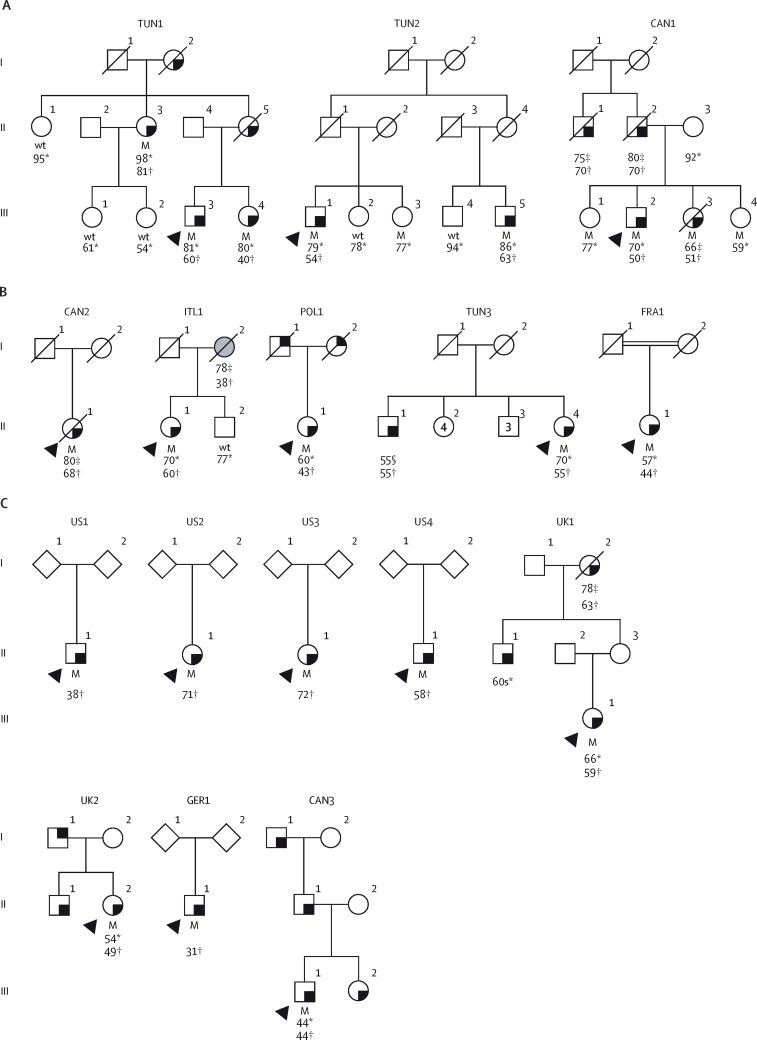
*RAB32* Ser71Arg co-segregates with Parkinson's disease (A) Simplified pedigrees for three families with the *RAB32* Ser71Arg mutation. (B) Subsequent genotyping and (C) Parkinson's disease database searches that identified affected *RAB32* Ser71Arg heterozygotes. Males are represented by squares and females by circles, numbers within those symbols refer to total counts, diamonds are where sex is undefined; probands are represented by a black arrowhead and a diagonal line indicates deceased individuals. People diagnosed with Parkinson's disease have a black square in the bottom right corner; the individual with a black square in the top right corner had reported tremor without a diagnosis; the person with a grey filled symbol had an unspecified progressive neurological disorder. Heterozygote mutant and wild-type genotypes are indicated with corresponding age at study analysis and age at symptom onset (if known). M=mutant. Wt=wild-type. *Age at study analyses. †Age at onset of the disease. ‡Age at death. §Lost to follow up.

A variant in the *RAB32* gene, c.213C>G (Ser71Arg; NCBI Reference Sequence: NM_006834.5; dbSNP: rs200251693) was identified in three probands, which cosegregated with parkinsonism in their pedigrees (figure 2A).^22^, ^23^ In the first pedigree from Tunisia (TUN1), a sib-pair (III-3 and III-4) and their aunt (II-3) were heterozygous for *RAB32* Ser71Arg, and presented with resting tremor at ages 60, 40, and 81 years, respectively (table 3). Three family members in TUN1 without signs of parkinsonism (III-1, III-2, and II-1) did not carry the mutation. In the second pedigree from Tunisia (TUN2), a first-cousin pair (III-1 and III-5) were both heterozygous for *RAB32* Ser71Arg. They initially presented with resting tremor at the ages of 54 years and 63 years, respectively. During their last visit, both individuals exhibited cognitive impairment, as evidenced by Mini-Mental State Examination (MMSE) scores of 17. Additionally, III-3 (sister of III-1 and first-cousin of III-5), who was asymptomatic at age 77 years, also carried *RAB32* Ser71Arg, whereas two additional unaffected family members (III-2 and III-4) did not. In the third pedigree from Canada (CAN1; of French origin), two affected siblings (III-2 and III-3) were heterozygous for *RAB32* Ser71Arg. The brother (III-2) was 70 years old and had presented with clumsiness on his left side and postural instability at age 50 years. He was diagnosed with akinetic-rigid Parkinson's disease. He had slowly progressive parkinsonism (Hoehn and Yahr stage 2) and mild cognitive impairment (Montreal Cognitive Assessment [MoCA] score of 26) after 17 years of disease. His affected sister (III-3) noticed pain in her right upper limb at age 51 years and died at age 66 years due to metastatic colorectal adenocarcinoma, at which time she had late-stage Parkinson's disease (Hoehn and Yahr stage 4). Additionally, two siblings (III-1 and III-4), who showed no signs of neurological disease at ages 77 years and 59 years, respectively, also carried *RAB32* Ser71Arg. No information on more subtle signs such as hyposmia, REM-sleep behaviour disorder, or orthostasis were available. Non-parametric linkage analysis was performed for all three families using their pedigree relationships, *RAB32* Ser71Arg genotyping results, and the frequency of the *RAB32* Arg71 allele in gnomAD=0·0000093 (15/1 613 550 alleles). This analysis showed the mutation and Parkinson's disease cosegregate (Z score=1·95; p=0·03) with a Kong and Cox logarithm of odds score LOD=1·51 at θ=0 (p=0·004). This is a statistical estimate of the relative probability that Parkinson's disease and the *RAB32* Arg71 allele are inherited together, and is significant for a single marker test.

**Table 3:** Clinical characteristics of RAB32 S71R heterozygotes

		**Country**	**Father's origin**	**Mother's origin**	**Sex**	**Age, years**	**Family history of parkinsonism**	**Age at onset, years**	**Disease duration, years**	**Initial symptom**	**Asymmetry at diagnosis**	**Diagnosis subtype**	**Cognition (MMSE score)**	**Positive response to levodopa**	**Other characteristics**	**Shared 0·36 Mb haplotype spanning *RAB32***
TUN1																
	III-3	Tunisia	Tunisia	Tunisia	Male	70	Yes	60	10	Resting tremor	NA	Tremor	29	Yes	NA	Yes
	II-3	Tunisia	Tunisia	Tunisia	Female	∼98	..	81	17	Resting tremor	NA	Tremor	15	Yes	NA	Yes
	III-4	Tunisia	Tunisia	Tunisia	Female	∼78	..	40	39	NA	NA	NA	21	Yes	NA	Yes
TUN2																
	III-1	Tunisia	Tunisia	Tunisia	Male	69	Yes	54	15	Resting tremor	NA	Tremor	17	Yes	NA	Yes
	III-5	Tunisia	Tunisia	Tunisia	Male	85	..	63	22	Resting tremor	NA	NA	17	Yes	NA	Yes
CAN1																
	III-2	Canada, Quebec	French-Canadian	NA	Male	70	Yes	50	21	Postural instability, clumsiness on left side	Yes	Akinetic-rigid	26 (MoCA after 17 years of disease)	Yes	Melanoma. Hyperlipidemia	Yes
	III-3	Canada, Quebec	French-Canadian	..	Female	66*	..	51	15	Pain right superior limb	Yes	NA	NA	Yes	Melanoma, died of colorectal carcinoma at 66 y	Yes
TUN3: II-1		Tunisia	Tunisia	Tunisia	Female	70	Yes, brother	55	16	Akinetic-ridged parkinsonism	Yes	Akinetic rigid	Normal	Yes, with drug-induced dyskinesia	Autonomic dysfunction (constipation and urinary), no oculomotor signs	Yes
CAN2: II-1		Canada, Saskatchewan	Romania	Germany	Female	80*	NA	68	12	Tremor (right leg), progressing to bradykinesia and rigidity within first year	Yes	Mixed	28 (normal)at 80 years	Amantadine, selegiline, before L-dopa after 9 years	Sparse neurofibrillary tangle pathology	Yes
POL1: II-1		USA	Poland	Poland	Female	60	Yes, both parents	43	17	Tremor	NA	Tremor	NA	NA	NA	Yes
ITL1: II-1		Canada, British Columbia	Italy	Italy	Female	70	Yes, mother unspecificed disease, onset 37 years	60	10	Bradykinesia	NA	NA	NA	Yes	NA	Yes
FRA1: II-1		France	Turkey	NA	Female	NA	No	44	NA	Tremor and bradykinesia	NA	NA	23 (mild cognitive impairment), 13 years post onset	Yes, but dyskinesia and dystonia	Autonomic dysfunction (urinary); high blood pressure and dyslipidemia	Yes
US1: II-1		USA	European	NA	Male	NA	Yes	38	NA	NA	NA	NA	NA	NA	NA	Yes
US2: II-1		USA	European	NA	Female	NA	Yes	71	NA	NA	NA	NA	NA	NA	NA	Yes
US3: II-1		USA	European	NA	Female	NA	Yes	72	NA	NA	NA	NA	NA	NA	NA	Yes
US4: II-1		USA	European	NA	Male	NA	No	58	NA	NA	NA	NA	NA	NA	NA	Yes
UK1: III-1		UK	European	NA	Female	66	Yes, maternal grandmother (onset 63, death 78), maternal uncle (onset 60s)	60	6	Right-sided rest tremor	Yes	Atypical	NA	Yes	NA	Yes
UK2: II-1		UK	European	NA	Female	54	Yes, father with tremor, brother with a similar presentation of left arm focal dystonia and polyneuropathy	49	40	Childhood dystonic tremor, upper limbs	Yes	Atypical	NA	NA	Hereditary demyelinating polyneuropathy, positive DaT-SPECT	Yes
GER1: II-1		Germany	NA	NA	Male	31	No	NA	NA	Right leg, then right upper limb tremor	Yes	Tremor	NA	Yes	Micrographia, hyposmia	NA
CAN3: III-1		Canada, Alberta	European	NA	Female	44	Yes, affected sister, father and paternal grandfather	44	NA	NA	NA	NA	NA	NA	NA	Yes

MoCA=Montreal Cognitive Assessment. MMSE=Mini-Mental State Examination. NA=not available. Conserved, shared 0·36 Mb haplotype spanning *RAB32.*

We subsequently genotyped *RAB32* Ser71Arg in population-matched individuals including 2604 unrelated people with Parkinson's disease (figure 1B). Of these, 2204 were people with Parkinson's disease of North American and European ancestry (mean age at onset 60·2 years [SD 11·8, range 26–80]; male to female ratio 1·7:1·0) and 400 were people with Parkinson's disease from Tunisia (mean age at onset 55·2 years [SD 14·7, range 13–87]; male to female ratio 1.0:1·0; appendix 2 p 2). Furthermore, 344 controls from Tunisia were included (mean age 66·3 [SD 11·1] years, range 39–100 years, male to female ratio 1·1:1·0; appendix 2 p 2). Five affected heterozygotes for *RAB32* Ser71Arg were identified (table 3, figure 2B). One affected proband identified, designated CAN2, was a female of Canadian origin with German and Romanian ancestry (II-1), who was diagnosed with Parkinson's disease at age 68 years and died 12 years later. She had onset of right leg tremor that progressed to include tremor, bradykinesia, and rigidity within 1 year. For the first 9 years, she had mild-to-moderate Parkinson's disease (Hoehn and Yahr stages 2–3) and was treated with dopamine agonists, amantadine, and selegiline. Subsequently, she benefited from levodopa without dyskinesia, but with mild wearing off symptoms. At age 80 years, at the last visit before she died, she was at Hoehn and Yahr stage 3 with no cognitive impairment (MMSE score 28). Neuropathological findings from this individual at autopsy showed mild-to-moderate neuronal loss in the substantia nigra pars compacta with sparse neurofibrillary tangles. Similar inclusion pathology was found in the thalamus and locus coeruleus. Mild-to-moderate globus pallidus neuronal loss was also observed, but ubiquitin and α-synuclein stains were unremarkable. A second affected proband, denoted the ITL1 pedigree, was a 70-year-old Canadian female (II-1) with parents of Italian ancestry who presented with bradykinesia at age 60 years. Her mother (I-2) had been bedbound for several years before her death at age 78 years, and had an unspecified progressive neurological disorder, with an age at onset of 37 years. A third affected proband, POL1, comprised a 60-year-old female (II-1) who was living in the USA; both parents were of Polish heritage. She was diagnosed with Parkinson's disease at age 43 years. Both her parents had tremor, but DNA samples were not available for testing. A third pedigree was identified from Tunisia, TUN3. The affected proband II-4 was a 70-year-old female who was diagnosed with Parkinson's disease at age 55 years. She had akinetic-rigid parkinsonism with good response to levodopa with drug-induced dyskinesia, as well as autonomic dysfunction including constipation and urinary dysfunction. No oculomotor abnormalities nor cognitive dysfunction were reported. Her brother (II-1) had developed Parkinson's disease at 55 years, but he was lost to follow-up a year later. He had akinetic-rigid parkinsonism and his first symptom was deterioration in gait. The fifth affected proband, denoted FRA1 (contributed by Sorbonne University, Paris, France) comprised a Turkish female (II-1) with no known family history of disease. She had slowness of movements and tremor at age 44 years. By age 57 years, her symptoms had slowly progressed to include bradykinesia and rigidity (Hoehn and Yahr stage 2), and an MMSE score of 23 after 13 years of disease duration. She developed non-motor signs, and symptoms including urinary disturbance. Levodopa treatment led to a considerable improvement of clinical signs, but she developed dyskinesia and dystonia.

Eight *RAB32* Ser71Arg heterozygotes were subsequently identified by bioinformatic analysis across four clinicogenomic databases (Figure 1, Figure 2, table 3), of whom four individuals of White, North American, or European descent with parkinsonism were identified via the AMP-PD database (US1, US2, US3, and US4). Notably, II-1 from US1 (age at onset of 38 years), II-1 from US2 (age at onset of 71 years), and II-1 from US3 (age at onset of 72 years) all had a positive family history for Parkinson's disease, whereas II-1 from US4 (age at onset of 58 years) was a sporadic case. Unfortunately, no additional clinical details were available. A total of 19 coding substitutions were identified in *RAB32* in the AMP-PD dataset (appendix 2 p 4), including two stop mutations, but only Ser71Arg was associated with Parkinson's disease (unadjusted χ^2^ test 4·9, p=0·026). Two people in the 100 000 Genomes Project database were heterozygous for *RAB32* Ser71Arg. The first, designated III-1 in the UK1 pedigree, was a 66-year-old British female of European ancestry. She was diagnosed with Parkinson's disease at age 60 years. Her symptoms began with a right-sided rest tremor 1 year before her diagnosis. She was levodopa-responsive and developed dyskinesia and dystonia of the right leg. Her maternal grandmother (I-2) had Parkinson's disease (age at onset of 63 years) and died at age 78 years, and her maternal uncle (II-1) had Parkinson's disease in his 60s. The second individual identified from the 100 000 Genomes Project was a 54-year-old female (II-2 in the UK2 pedigree) of European ancestry. Her symptoms began in adolescence with a dystonic tremor affecting her upper limbs. She had features suggestive of a polyneuropathy that was later shown to be a hereditary demyelinating neuropathy with neurophysiology studies. She developed parkinsonism and was diagnosed with Parkinson's disease at age 49 years. DaT-SPECT imaging showed a profound dopaminergic reduction of tracer uptake in the left caudate nucleus. MRI showed non-specific white matter changes and her CSF was unremarkable. Her brother (II-1) had a similar clinical workup and developed early onset parkinsonism with dystonia. Her father (I-1) had tremor but was never diagnosed with Parkinson's disease. 20 variants in *RAB32* were identified via the 100 000 Genomes Project (appendix 2 p 4). Another pedigree (GER1) was identified in a private database for clinical genome and exome sequencing (University of Tübingen, Tübingen, Germany). This individual was male (II-1) and had reported difficulties with his right leg when jogging at age 31 years and had genetic testing. Subsequently, he noticed tremor in his right upper limb and micrographia. Neurological examination documented mild right-sided bradykinesia and rigor, mild bilateral resting tremor, abnormal gait with reduced right arm swing, and hyposmia. A likely diagnosis of levodopa-responsive early-onset Parkinson's disease was made. In the same database, three other young female heterozygotes were identified with non-neurological disorders. Their symptoms included abnormal inflammatory responses, proteinuria (a nephropathy consisting of focal segmental glomerulosclerosis), and Tetralogy of Fallot with pulmonary stenosis. Diagnostic testing was done at age 22 years in one of these individuals and at age 15 years in the other two individuals, but no other clinical nor genetic data were available. Lastly, one female with *RAB32* Ser71Arg was identified through the GP2 database (III-1 in CAN3). She was from Alberta, Canada, but was ancestrally European, with an age at onset of 44 years and family history of parkinsonism, including her sister (III-2), father (II-1), and paternal grandfather (I-1). No other clinical data were available. Whole genome sequence data in GP2 showed nine coding variants in *RAB32*, but there were too few control participants to enable association analysis (appendix 2 p 4).

Next, we examined the association of *RAB32* Ser71Arg with Parkinson's disease risk in each case–control series (table 4). While results were equivocal in data from Tunisia (p>0.99) and in the GP2 database (p>0·99), as relatively few controls were included, the findings were significant in the AMP-PD database (p=0·044) and the UK 100 000 Genomes Project (p=0·0005; table 4). Subsequent meta-analysis across all series confirmed a significant effect (OR 13·17, 95% CI 2·15–87·23).

**Table 4:** Association analyses in case–control datasets

		**Cases**	**Controls**	Odds ratio (95% CI)*	**p value**
Case–control studies sample set					
	Tunisia	1/400 (0·25%)	0/344	2·59 (0·11–63·71)	>0·99
	North American	4/2204 (0·18%)	NA	NA	NA
	AMP-PD	4/3105 (0·13%)	0/3670	10·65 (0·57–197·91)	0·044
	GP2	1/1849 (0·05%)	0/349	0·57 (0·02–13·96)	>0·99
	German clinical diagnostic database	1/311 (0·32%)	3/23389 (0·01%)	32·28 (4·75–219·33)	0·052
	100 000 Genomes Project	2/778 (0·26%)	0/35141	226·28 (10·85–4717·48)	0·0005
Meta-analysis		13/8717 (0·15%)	3/65693 (<0·01%)	13·17 (2·15–87·23)†	0·0055

Database resources were last accessed between Sept 1 and Dec 20, 2023. AMP-PD=Accelerating Medicines Partnership in Parkinson's Disease. GP2=Global Parkinson's Genetics Program. NA=None or not applicable.

* When zeros create computation challenges for the odds ratios or its SE, a constant (0·5) was added to all cells as a continuity correction.

† The null hypothesis that *RAB32* Ser71Arg confers no risk is rejected (Z score=2·77 p<0·006; Q_(df=5)_=9647, p=0·0001; *I*^2^=99·96%).

Haplotype analysis established gametic phase for the *RAB32* locus at chromosome 6q24.3 within three pedigrees (TUN1, TUN2, and CAN1; figure 1A). Genotypes of nine SNPs adjacent to *RAB32* c.213C>G showed that 15 of 16 probands shared a 332 413–384 587 base pair ancestral haplotype that appeared to be inherited identical-by-descent (table 3; appendix 2 p 5).

Biological analysis of *RAB32* mRNA and protein expression in various tissue samples obtained from people in publicly available databases showed a ubiquitous but low level of expression (figure 1E; appendix 1 p 9). Although expression was highest in non-brain tissues, such as immune cells and lung, brain expression was observed in spinal cord and substantia nigra pars compacta. Analyses with the Human Protein Atlas confirmed RAB32 protein expression in endothelial cells, neuronal cells, and glia (appendix 1 p 9). Subsequent immunohistochemical analysis revealed endogenous RAB32 expression within murine dopaminergic neurons (appendix 1 p 9).

Structural modelling suggests the RAB32 Ser71Arg substitution is pathogenic, and is supported by conservation of the variant in orthologs (GERP score 4·68, CADD score 19·2; figure 1F; appendix 1 p 10). Structural modelling using AlphaFold predicted a robust interaction between RAB32 and LRRK2, similar to RAB29 and RAB38 (appendix 1 pp 11–12). Modelling indicated that RAB32 Arg71 would not affect LRRK2 binding (appendix 1 p 12). Indeed, transfected RAB32 co-immunoprecipitated with endogenous LRRK2, and the Arg71 mutation did not impair this interaction (figure 1G; appendix 1 p 13). Overexpression of wild-type RAB32 increased LRRK2-direct substrate phosphorylation without enhancing LRRK2 autophosphorylation (Tukey's multiple test comparison q_(15)=_16·4 p<0·0001 and q_(15)_=0·05 p>0·99, respectively; appendix 1 p 14). Expression of RAB32 Arg71 enhanced LRRK2-direct substrate phosphorylation and LRRK2 autophosphorylation more than RAB32 Ser71 (Tukey's q_(15)_=44 p<0·0001 and q_(15)_=13·5 p<0·0001, respectively). RAB32 Arg71 induced LRRK2 autophosphorylation to a lower level than observed with the positive control, RAB29 (RAB32 Arg71 *vs* RAB29 for LRRK2-direct substrate phosphorylation Tukey's q_(15)_=12·3 p<0·0001 and LRRK2 autophosphorylation q_(15)_=36·5 p<0·0001, respectively; appendix 1 p 14). Consistent with increased LRRK2 activity, RAB32 Arg71 reduced phosphorylation at this biomarker site more than RAB32 Ser71 (Tukey's q_(15)_=5·7 p<0·009), comparable to RAB29 (Tukey's q_(15)_=2·3 p=0·52). In contrast, RAB32 Ala71 functioned like RAB32 Ser71 and did not enhance LRRK2 activation (appendix 1 p 14).

Lastly, we examined the potential interaction between RAB32 and PINK1. Confocal microscopy of HEK293 cells transiently cotransfected with mCherry-Parkin, GFP-RAB32, and HA-PINK1 revealed significant co-localization of GFP-RAB32 with HA-PINK1 but not with mCherry-Parkin (appendix 1 p18). Colocalisation with PINK1 was significantly reduced in cells transfected with RAB32 Arg71 compared to Ser71, and was reduced to a similar extent as kinase dead PINK1 (KD-PINK1; appendix 1 p 18). Cells transfected with both RAB32 Arg71 and KD-PINK1 showed the lowest colocalisation (appendix 1 p18). Our results suggest that both PINK1 kinase activity and RAB32 Ser71 are important for PINK1-RAB32 colocalisation.

## Discussion

The findings of our genetic and clinical analyses showed that the *RAB32* variant Ser71Arg cosegregates with Parkinson's disease in three families; an additional 13 unrelated heterozygotes were identified in case–control analyses. All affected people had a clinical diagnosis of levodopa-responsive Parkinson's disease, with a mean age at onset of 54·6 years (SD 12·75, range 31–81), and two-thirds had a known family history of parkinsonism. These findings are comparable with those in people with Parkinson's disease with mutations in *LRRK2* (mean age at onset of 58·2 years [SD 12]) or *VPS35* (mean age at onset of 56·5 years [SD 12]).^24^ In most of our *RAB32* Ser71Arg heterozygotes, tremor was the initial symptom, and Parkinson's disease onset, clinical variability, and progression were consistent with typical late-onset Parkinson's disease.^25^ The cases of melanoma (in the CAN1 and ITL1 pedigrees) and polyneuropathy (in the UK1 pedigree) could be incidental. The *RAB32* Ser71Arg variant was also identified in three asymptomatic family members, one in TUN2 (aged 77 years at the time of the study) and two in CAN1 (aged 51 and 77 years at the time of the study), and the variant had a non-negligible frequency in gnomAD that suggests penetrance is incomplete. Although incomplete penetrance is typical for monogenic forms of Parkinson's disease, and consistent with a disease mechanism that activates LRRK2 kinase, penetrance estimates also depend on how carefully non-manifesting heterozygotes are characterised. Therefore, studies on dopaminergic dysfunction in clinically asymptomatic heterozygotes, along with more comprehensive prodromal testing, will be important.

Given the disparate geographical origin of the identified *RAB32* Ser71Arg heterozygotes, it is remarkable that only one Arg71 haplotype was observed (appendix 2, p 5), suggesting that all probands who were genotyped originate from one ancestral founder, reminiscent of *LRRK2* Gly2019Ser.^16^

Neuropathological findings in II-1 from CAN2 revealed mild-to-moderate neuronal loss and neurofibrillary tangle inclusions in the substantia nigra, with no Lewy body pathology. Almost half of *LRRK2* cases examined at autopsy have dopaminergic neuronal loss with gliosis, without the presence of α-synuclein, which suggests this intracellular inclusion pathology is a secondary phenomenon.^26^

Our initial in-vitro characterisation of the *RAB32* variant Ser71Arg showed that the Arg71 mutant protein does not disrupt the binding of RAB32 to LRRK2 (appendix 1 pp 12–13), but rather stimulates LRRK2 kinase activity (appendix 1 p 14). This interaction is likely to be mediated by a specific LRRK2–RAB interaction domain (known as “site-1”)^18^ because RAB32 Arg71 is no longer able to activate LRRK2 when site-1 is mutated to Leu403 (appendix 1 p 16). Despite these observations, how RAB32 Arg affects the ability of LRRK2 to form an active conformation remains to be determined.

RAB32 is expressed in multiple human tissues, including immune cells and dopaminergic neurons of the brain (appendix 1, p 9). Pathogenic mutations in LRRK2 lead to hyper-phosphorylation of a subset of RAB GTPases proteins, and our in vitro studies suggest that RAB32 Arg71 might lead to a similar hyper-phosphorylated state of RAB GTPases (appendix 1, pp 14-17). This in turn, can stall endosomal and lysosomal trafficking, and accelerate the accumulation of intracellular misfolded proteins leading to dopaminergic cell loss and the activation of immune cells, two processes involved in disease progression in Parkinson's disease.

Our study has limitations. Although, several rare variants were identified in RAB GTPases, including many in *RAB32* (appendix 2 pp 3, 4), the power of our sample size was limited for burden testing of all variants in RAB GTPases. Future studies will require much larger case series with matched controls. Nevertheless, *RAB32* Ser71Arg was observed in three affected probands and led us to test the a priori hypothesis of linkage analysis for a single marker, rather than genome-wide. Although, our sample size was sufficient for rare variant meta-analysis, genome-wide genotyping to match cases with controls could help correct for potential population stratification. To the best of our knowledge, only one individual with the *RAB32* Ser71Arg variant has come to autopsy, with sparse neurofibrillary tangle pathology in the midbrain and thalamus, without Lewy body pathology. Similar post-mortem observations have been made in *LRRK2* and *PINK1* parkinsonism, but further comparisons would be helpful. Our functional studies are limited to in-vitro assays that overexpress mutant proteins, so additional studies in knock-in rodent and patient-derived samples are needed for validation.

*RAB32* Ser71Arg most likely has an important role in the pathogenesis of Parkinson's disease. Our results show that RAB32 interacts with LRRK2, and that the *RAB32* Ser71Arg pathogenic variant activates LRRK2 kinase, reminiscent of *LRRK2* and *VPS35* mutations. RAB32 also interacts with known retromer binding partner, VARP,^27^ and can colocalise with PINK1 (appendix 1 p 18). However, RAB32 is best described to mediate mitochondrial and lysosome-related pathways,^28^ and it is unlikely to be a coincidence that missense mutations in *LRRK2, VPS35*, and *RAB32* all constitutively activate LRRK2 kinase activity. While each gene linked to Parkinson's disease compromises the age-associated viability of dopaminergic neurons in the substantia nigra, RAB32 indicates that several of these proteins might have a more intimate synergistic relationship to control pathogen growth in immune cells.^29^ Further study in different cellular contexts, of cell autonomous and non-autonomous effects, is imperative. Given the shared ancestral haplotype of affected probands with *RAB32* Ser71Arg, and their dispersed geographical origin, we anticipate more heterozygotes with this pathogenic variant can be identified through genotyping array data that has already been generated in GWAS of Parkinson's disease.^1^ The population origin and frequency of those heterozygotes, their genealogic data, affection status, and associated clinical findings might readily inform penetrance estimates, prognosis, and genetic counselling and should be pursued.

## Data sharing

Exome and array data on the families used in this study are available via the corresponding author, subject to terms on data sharing in their original consents. All GP2 data are hosted in collaboration with the Accelerating Medicines Partnership in Parkinson's Disease (AMP-PD) and are available via application on the AMP-PD website. All Genomics England data are hosted in a cloud. To access the data, researchers must first apply to become a member of either the Genomics England Clinical Interpretation Partnership (academics, students, and clinicians) or the Discovery Forum (industry partners). All AMP-PD data are available through https://www.amp-pd.org/ and require access approval. Data from the German clinical diagnostic database will be made available on request to manu.sharma@uni-tuebingen.de.

## Declaration of interests

AR receives unrestricted research support from the Dr Ali Rajput Endowment for Parkinson's Disease and Movement Disorders; in the past 2 years AR has received honoraria from Quebec Consortium for Drug Discovery–Brain Canada and Ipsen Biopharmaceuticals Canada. MSG reports grants from National Institutes of Health (NIH)–National Institute of Neurological Disorders and Stroke (NINDS) and the Michael J Fox Foundation for Parkinson's Research. AJS has received fees from Neurocrine (Chair, Data and Safety Monitoring Board [DSMB]), AskBio (Member, DSMB) and Capsida (advisor), receives a stipend from the International Parkinson's and Movement Disorders Society (Editor-in-Chief, *Movement Disorders*) and grant funding from Michael J Fox Foundation, Weston Brain Institute, and Brain Canada. ZG-O received consultancy fees from Bial Biotec, Bial, Capsida, Handl Therapeutics, Idorsia, Neuron23, Ono Therapeutics, Prevail Therapeutics, UCB, and Vanqua. He reports grants from the Michael J Fox Foundation for Parkinson's Research, The Weston Family Foundation, The Silverstein Foundation, NIH, and the Canadian Consortium on Neurodegeneration in Aging. HRM is employed by University College Londn. In the last 12 months he reports paid consultancy from Roche, Aprinoia, AI Therapeutics, and Amylyx; lecture fees or honoraria from BMJ, Kyowa Kirin, Movement Disorders Society; and research grants from Parkinson's UK, Cure Parkinson's Trust, PSP Association, Medical Research Council, and Michael J Fox Foundation. HRM is a co-applicant on a patent application related to C9ORF72: method for diagnosing a neurodegenerative disease (PCT/GB2012/052140). MJF reports US patents associated with *LRRK2* mutations and mouse models (8409809 and 8455243), and methods of treating neurodegenerative disease (20110092565). SA-C has received honoraria from Merz, and grant funding from the Pacific Parkinson's Research Institute, the Weston Family Foundation, Parkinson Canada, Canadian Institutes of Health Research (CIHR), the VGH and UBC Hospital Foundation, Rick's Heart Foundation, and the Jack and Darlene Poole Foundation. All other authors declare no competing interests.
